# Supplementary material for: Treatment of monogenic and digenic dominant genetic hearing loss by CRISPR-Cas9 ribonucleoprotein delivery in vivo
Source: Nat Commun. 2023 Aug 15;14:4928. doi: 10.1038/s41467-023-40476-7 (PMC10427623; doi:10.1038/s41467-023-40476-7)
Supplement: Supplementary file 1 — Supplementary Information [file 41467_2023_40476_MOESM1_ESM.pdf]

Supplementary Fig. 1.

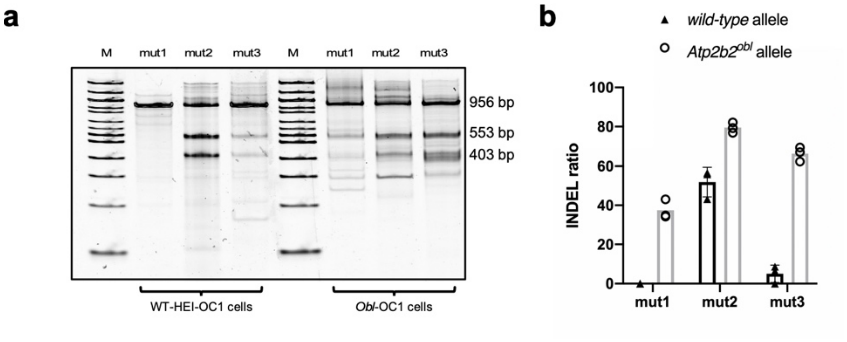

c

Wildtype OC1 cells, SpCas9/Atp2b2-mut1

5'-TCATCATGGACACGTTTGCCTCCCTGGCCCTGGCCACAGAGCCACCTACGGAGACTCTGC -3'	Wild type reference	**Reads**	**Ratio**
5'-TCATCATGGACACGTTTGCCTCCCTGGCCCTGGCCACAGAGCCACCTACGGAGACTCTGC -3'	Non-edited	58128	100%

Wildtype OC1 cells, SpCas9/Atp2b2-mut2

5'-TCATCATGGACACGTTTGCCTCCCTGGCCCTGGCCACAGAGCCACCTACGGAGACTCTGC -3'	Wild type reference	**Reads**	**Ratio**
5'-TCATCATGGACACGTTTGCCTCCCTGGCCCTGGCCACAGAGCCACCTACGGAGACTCTGC -3'	Non-edited	6749	41.6%
5'-TCATCATGGACACGTTTGCCTCCC - - - - - - -TGGCCACAGAGCCACCTACGGAGACTCTGC -3'	-6 bp	5523	34.0%
5'-TCATCATGGACACGTTTGCCTCCCTG -CCCTGGCCACAGAGCCACCTACGGAGACTCTGC -3'	-1 bp	2728	16.8%
5'-TCATCATGGACACGTTTGCCTCC - - - - CCCTGGCCACAGAGCCACCTACGGAGACTCTGC -3'	-4 bp	221	1.4%
5'-TCATCATGGACACGTTTGCCTCCCT - - CCCTGGCCACAGAGCCACCTACGGAGACTCTGC -3'	-2 bp	221	1.4%
5'-TCATCATGGACACGTTTGCCTC - - - - - -CCCTGGCCACAGAGCCACCTACGGAGACTCTGC -3'	-5 bp	356	2.2%
5'-TCATCATGGACACGTTTGCCTCCC - - - -CCCTGGCCACAGAGCCACCTACGGAGACTCTGC -3'	-3 bp	155	1.0%

Wildtype OC1 cells, SpCas9/Atp2b2-mut3

5'-TCATCATGGACACGTTTGCCTCCCTGGCCCTGGCCACAGAGCCACCTACGGAGACTCTGC -3'	Wild type reference	**Reads**	**Ratio**
5'-TCATCATGGACACGTTTGCCTCCCTGGCCCTGGCCACAGAGCCACCTACGGAGACTCTGC -3'	Non-edited	73715	93.8%
5'-TCATCATGGACACGTTTG - - - - - - - - - - - CCCTGGCCACAGAGCCACCTACGGAGACTCTGC -3'	-9 bp	566	0.7%
5'-TCATCATGGACACGTTTGCCTCCCT -GCCCTGGCCACAGAGCCACCTACGGAGACTCTGC -3'	-1 bp	527	0.4%
5'-TCATCATGGACACGTTTGCCTCCCTGTGCCCTGGCCACAGAGCCACCTACGGAGACTCTGC -3'	+1 bp	3357	0.7%
5'-TCATCATGGACACGTTTGCCTCCCT - - CCCTGGCCACAGAGCCACCTACGGAGACTCTGC -3'	-2 bp	108	0.1%

Obl-OC1 cells, SpCas9/Atp2b2-mut1

5'-TCATCATGGACACGTTTGCCTTCCTGGCCCTGGCCACAGAGCCACCTACGGAGACTCTGC -3'	Obl reference	**Reads**	**Ratio**
5'-TCATCATGGACACGTTTGCCTTCCTGGCCCTGGCCACAGAGCCACCTACGGAGACTCTGC -3'	Non-edited	3623	65.2%
5'-TCATCATGGACACGTTTGCCT -CCTGGCCCTGGCCACAGAGCCACCTACGGAGACTCTGC -3'	-1 bp	1935	34.8%

Obl-OC1 cells, SpCas9/Atp2b2-mut2

5'-TCATCATGGACACGTTTGCCTTCCTGGCCCTGGCCACAGAGCCACCTACGGAGACTCTGC -3'	Obl reference	**Reads**	**Ratio**
5'-TCATCATGGACACGTTTGCCTTCCTGGCCCTGGCCACAGAGCCACCTACGGAGACTCTGC -3'	Non-edited	11198	34.4%
5'-TCATCATGGACACGTTTGCCTTCCTGG- CCTGGCCACAGAGCCACCTACGGAGACTCTGC -3'	-1 bp	7044	13.3%
5'-TCATCATGGACACGTTTGCCTTCCTGG- - CTGGCCACAGAGCCACCTACGGAGACTCTGC -3'	-2 bp	4032	7.6%
5'-TCATCATGGACACGTTTGCCTTCCTG - - - CTGGCCACAGAGCCACCTACGGAGACTCTGC -3'	-3 bp	3302	2.3%
5'-TCATCATGGACACGTTTGCCTTC - - - - - GCTGGCCACAGAGCCACCTACGGAGACTCTGC -3'	-5 bp	4581	0.7%
5'-TCATCATGGACACGTTTGCCTTCCTGGACCCTGGCCACAGAGCCACCTACGGAGACTCTG -3'	+1 bp	276	1.2%
5'-TCATCATGGACACGTTTGCCTTC - - - - - - - - - GGCCACAGAGCCACCTACGGAGACTCTGC -3'	-8 bp	2133	1.8%

Obl-OC1 cells, SpCas9/Atp2b2-mut3

5'-TCATCATGGACACGTTTGCCTTCCTGGCCCTGGCCACAGAGCCACCTACGGAGACTCTGC -3'	Obl reference	**Reads**	**Ratio**
5'-TCATCATGGACACGTTTGCCTTCCTGGCCCTGGCCACAGAGCCACCTACGGAGACTCTGC -3'	Non-edited	20235	37.7%
5'-TCATCATGGACACGTTTGCCTTCCTTGGCCCTGGCCACAGAGCCACCTACGGAGACTCTG -3'	+1 bp	26437	49.3%
5'-TCATCATGGACACGTTTGCCTTCCT- GCCCTGGCCACAGAGCCACCTACGGAGACTCTGC -3'	-1 bp	2516	4.7%
5'-TCATCATGGACACGTTTGCCTTCC- - - - - - -TGGCCACAGAGCCACCTACGGAGACTCTGC -3'	-6 bp	821	1.5%
5'-TCATCATGGACACGTTTGCCTTCCT - -CCCTGGCCACAGAGCCACCTACGGAGACTCTGC -3'	-2 bp	981	1.8%
5'-TCATCATGGACACGTTTGCCTTCCTTGGGCCCTGGCCACAGAGCCACCTACGGAGACTCT -3'	+2 bp	945	1.8%
5'-TCATCATGGACACGTTTGCCTTCC - - -CCCTGGCCACAGAGCCACCTACGGAGACTCTGC -3'	-3 bp	441	1.8%
5'-TCATCATGGACACGTTTGCCTTCC - - - - - - - GGCCACAGAGCCACCTACGGAGACTCTGC -3'	-7 bp	435	0.8%
5'-TCATCATGGACACGTTTGCCTTCC - - - - - CTGGCCACAGAGCCACCTACGGAGACTCTGC -3'	-5 bp	591	0.8%
5'-TCATCATGGACACGTTTGCCTTCC - - - - - - - - - - - CACAGAGCCACCTACGGAGACTCTGC -3'	-10 bp	231	0.4%

Allele-selective editing of WT or *Obl* mutant *Atp2b2* gene in OC1 cell line. (**a**) T7E1 assay of the DNA from the HEI-OC1 and Obl-OC1 cells nucleofected with RNP complexes. The two resulting DNA fragments from cleavage were indicated by asterisks. Gel were repeated independently for 3 time with similar results. (**b**) Distribution of the indels by NGS from the HEI-OC1 and Obl-OC1 cells nucleofected with RNP complexes. Columns and error bars represent the mean ± SD. n=3 biologically independent experiments. (**c**) NGS analysis identified indel reads in the WT HEI-OC1 and Obl-OC1 cells nucleofected with three RNP complexes. The arrow points to the major editing event of 1 bp deletion by *Atp2b2*-Mut1 in the Obl-OC1 cells.

.

Supplementary Fig. 2.

a

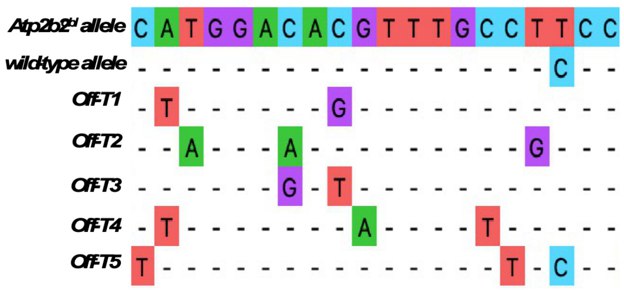

b

	5'-Sequence-3'	Mismatches (MMs)	NCBI accession	Location
Atp2b2^{obl}	CATGGACACGTTTGCCTTCCTGG	0MMs	N/A	chr6
Off-T1	CTTGGACAGGTTTGCCTTCCAGG	2MMs[2:9]	AC160121.2	chr13
Off-T2	CAAGGAAACGTTTGCCTGCCTGG	3MMs[3:7:18]	AC121297.20	chr1
Off-T3	CATGGAGATGCTTGCCTTCCAGG	2MMs[7:9]	AC165258.2	chr17
Off-T4	CTTGGACACATTTGTCTTCCAGG	3MMs[2:10:15]	AC102291.7	chr15
Off-T5	TATGGACACGTTTGCTTCCCTGG	3MMs[1:16:18]	XM_030255153.1	chr1

c

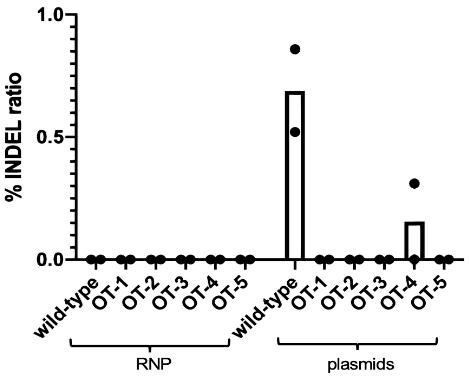

d

e

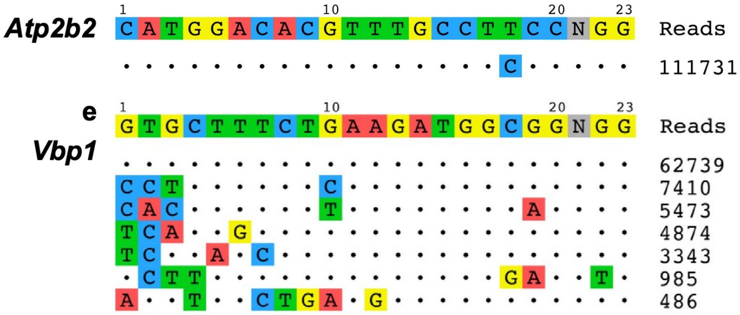

f

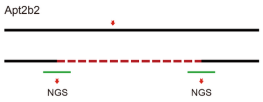

Apt2b2 exon24, -997-1010 intronic sites

5'-TGGAGCTCCCTCTAGTGGTGGTTTATAGACATCGTGTGACAGCTTCCTGGAGACTGGACAGATGCCAGTTAAGAAAACAGGTTTG -3' Wild type reference **Reads**

5'-TGGAGCTCCCTCTAGTGGTGGTTTATAGACATCGTGTGACAGCTTCCTGGAGACTGGACAGATGCCAGTTAAGAAAACAGGTTTG -3' WT injected 80665 (99.85%)

5'-TGGAGCTCCCTCTAGTGGTGGTTTATAGACATCGTGTGACAGCTTCCTGGAGACTGGACAGATGCCAGTTAAGAAAACAGGTTTG -3' Apt2b2$^{Obl/+}$ injected 75771(99.85%)

Apt2b2 exon24, +661-821 intronic sites

5'-AGGCAAAGGGAAGAAACATTGCTCGCTTCATGGCCAGGAAGCAGAGAAAAGTATGGACCAGGCCCCACAGTTCCCTTCAAGAGAA -3' Wild type reference **Reads**

5'-AGGCAAAGGGAAGAAACATTGCTCGCTTCATGGCCAGGAAGCAGAGAAAAGTATGGACCAGGCCCCACAGTTCCCTTCAAGAGAA -3' WT injected 67534 (100%)

5'-AGGCAAAGGGAAGAAACATTGCTCGCTTCATGGCCAGGAAGCAGAGAAAAGTATGGACCAGGCCCCACAGTTCCCTTCAAGAGAA -3' Apt2b2$^{Obl/+}$ injected 66607 (100%)

Off-target analysis by computational prediction and GUIDE-seq. (**a)** Editing efficiency of predicted off-target was analyzed by NGS in Obl-OC cells transfected with RNP or plasmid DNA using a LONZA 4D-Nucleofector. (**b**) WT allele and five off-target sites were identified by computational prediction using the CRISPR Design Tool (*28*). Mismatch positions are indicated counting the PAM as positions 21-23. None of these loci were associated with hearing function. **(c)** Editing efficiency of predicted off-target loci analyzed by NGS in Obl-OC cells transfected with either RNP or plasmid DNA using a LONZA 4D-Nucleofector. The RNP delivery did not yield any off-target effect whereas the plasmid delivery showed low-frequency editing at the WT and OT-4 loci. (**d**) Editing efficiency of predicted off-target loci analyzed by GUIDE-seq in Obl-OC cells transfected with Cas9:Atp2b2-mut1 RNP. No off-target loci were observed by GUIDE-seq method. (**e**) GUIDE-seq study on control gene Vbp1sgRNA in Obl-OC1 cells by RNP delivery identified multiple off-targets with varying frequencies shown by NGS. Mismatches compared to the on-target site are shown and highlighted in color. (**f**) NGS analysis on the intronic sites of large deletion junctions from injected $Apt2b2^{Obl/+}$ or $Apt2b2^{+/+}$ cells. No indels were detected from the junction site.

Supplementary Fig. 3.

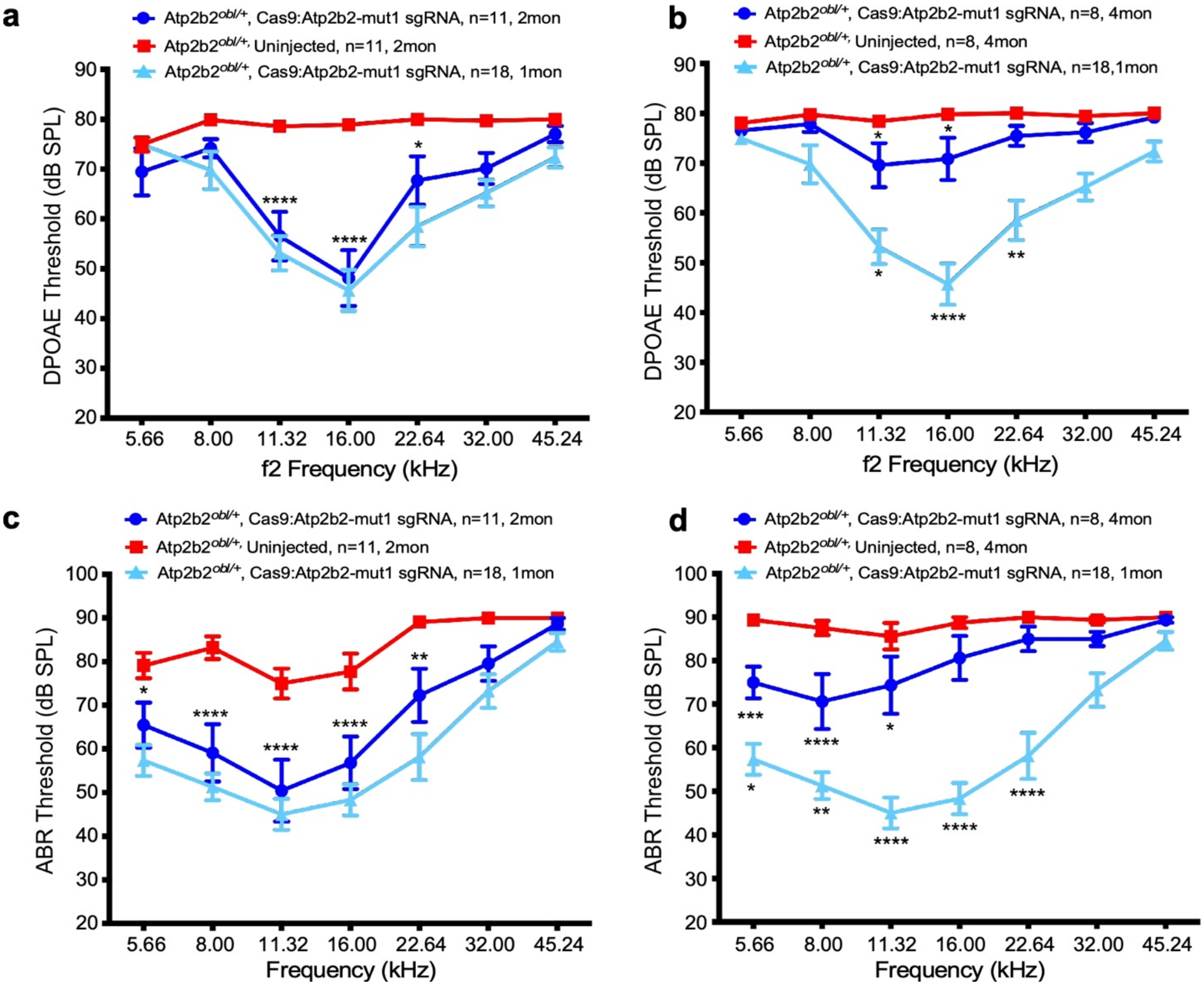

The rescue of OHC function and hearing showed diminished effect over time by *in vivo* lipid mediated delivery of Cas9:sgRNA. (**a**). Two months post injection, significantly reduced DPOAE thresholds were detected in $Atp2b2^{Obl/+}$ ears injected with Cas9:Atp2b2-mut1:Lipo2000 (blue) compared to uninjected $Atp2b2^{Obl/+}$ ears (red). The DPOAE threshold reduction was comparable to that at one month post injection (cyan). (**b**). Four months post injection, significantly reduced DPOAE thresholds were detected at two frequencies (11.32 and 16 kHz) in $Atp2b2^{Obl/+}$ ears injected with Cas9:Atp2b2-mut1:Lipo2000 (blue) compared to uninjected $Atp2b2^{Obl/+}$ ears (red). Compared to DPOAE thresholds one month post injection (cyan), the rescue effect has greatly diminished at three frequencies (11.32, 16 and 22.64 kHz). (**c**)**.** Two months post injection, significantly reduced ABR thresholds were detected in $Atp2b2^{Obl/+}$ ears injected with Cas9:Atp2b2-mut1:Lipo2000 (blue) compared to uninjected $Atp2b2^{Obl/+}$ ears (red). The ABR threshold reduction was comparable to that at one month post injection (cyan). (**d**). Four months post injection, significantly reduced ABR thresholds were detected at three frequencies (5.66, 8 and 11.32 kHz) in $Atp2b2^{Obl/+}$ ears injected with Cas9:Atp2b2-mut1:Lipo2000 (blue) compared to uninjected $Atp2b2^{Obl/+}$ ears (red). Compared to ABR thresholds one month post injection (cyan), the rescue effect has greatly diminished at five frequencies below 32 kHz. Statistical tests were

two-way ANOVA with Bonferroni correction for multiple comparisons: $^{**}p < 0.01$, $^{***}p < 0.001$, and $^{****}p < 0.0001$. Values and error bars reflect mean ± SEM.

Supplementary Fig. 4.

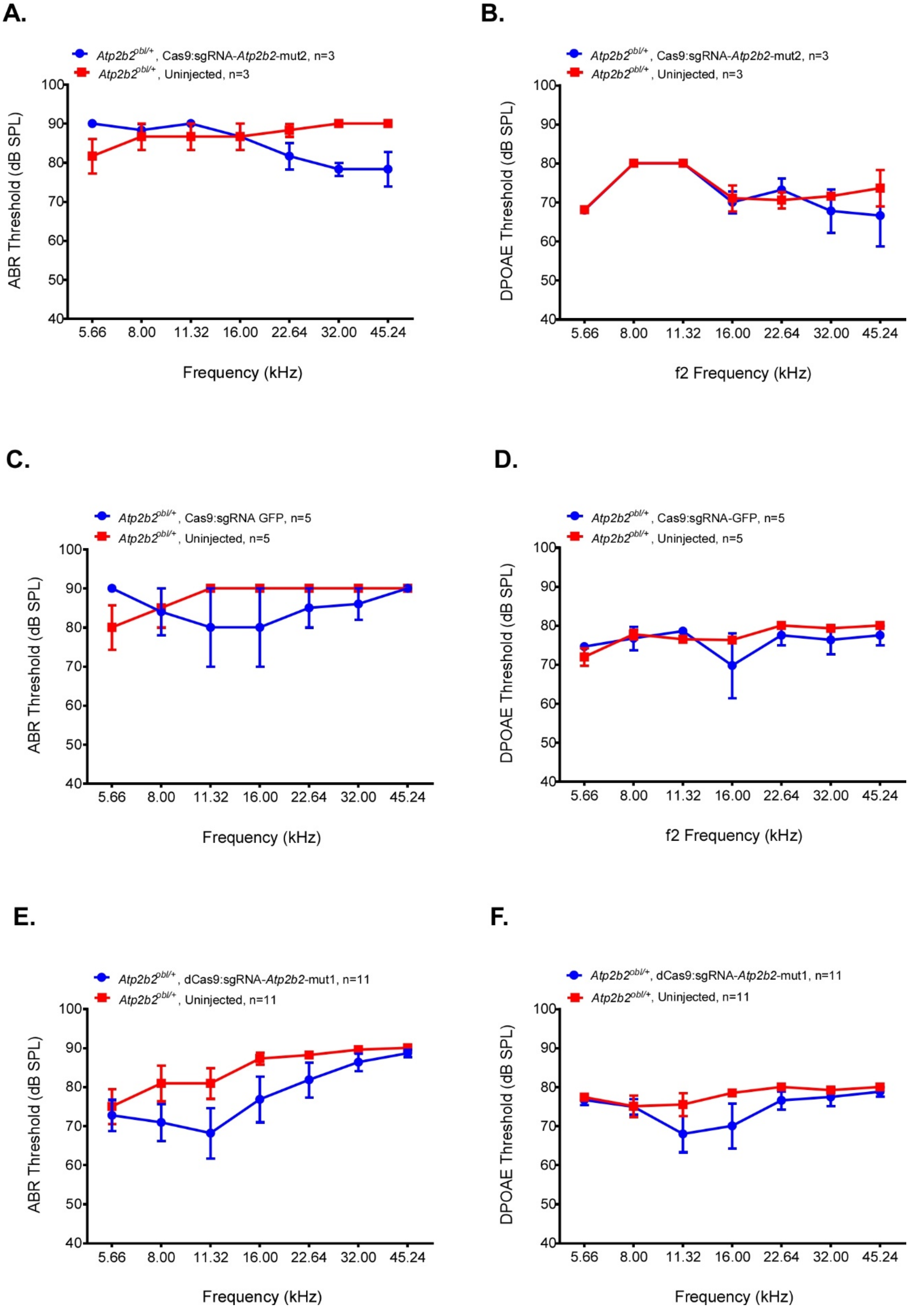

Hearing rescue is gRNA and Obl allele specific and depends on Cas9 DNA cleavage activity. (**A**) ABR and (**B**) DPOAE thresholds in Atp2b2$^{Obl/+}$ ears injected with Cas9:Atp2b2-mut2 sgRNA:Lipo2000 (blue) and uninjected Atp2ba2$^{Obl/+}$ ears (red) at four weeks. Values and error bars reflect mean ± SEM. (**C**) ABR and (**D**) DPOAE thresholds in Atp2b2Obl/+ ears injected with Cas9:GFP-sgRNA:Lipo2000 (blue) and uninjected Atp2b2$^{Obl/+}$ ears (red) at four weeks. Values and error bars reflect mean ± SEM. (**E**) ABR and (**F**) DPOAE thresholds in Atp2b2$^{Obl/+}$ ears injected with dCas9:Atp2b2-mut1 sgRNA:Lipo2000 (blue) and uninjected Atp2b2$^{Obl/+}$ ears (red) at four weeks. Values and error bars reflect mean ± SEM.
