# Supplementary material for: Treatment of monogenic and digenic dominant genetic hearing loss by CRISPR-Cas9 ribonucleoprotein delivery in vivo
Source: Nat Commun. 2023 Aug 15;14:4928. doi: 10.1038/s41467-023-40476-7 (PMC10427623; doi:10.1038/s41467-023-40476-7)
Supplement: Supplementary file 3 — Reporting Summary [file 41467_2023_40476_MOESM3_ESM.pdf]

nature portfolio

Corresponding author(s): Zheng-Yi Chen
Last updated by author(s): Jul 11, 2023

Reporting Summary

Nature Portfolio wishes to improve the reproducibility of the work that we publish. This form provides structure for consistency and transparency in reporting. For further information on Nature Portfolio policies, see our Editorial Policies and the Editorial Policy Checklist.

Statistics

For all statistical analyses, confirm that the following items are present in the figure legend, table legend, main text, or Methods section.

n/a	Confirmed	
☐	☒	The exact sample size (n) for each experimental group/condition, given as a discrete number and unit of measurement
☐	☒	A statement on whether measurements were taken from distinct samples or whether the same sample was measured repeatedly
☐	☒	The statistical test(s) used AND whether they are one- or two-sided<br>*Only common tests should be described solely by name; describe more complex techniques in the Methods section.*
☐	☒	A description of all covariates tested
☐	☒	A description of any assumptions or corrections, such as tests of normality and adjustment for multiple comparisons
☐	☒	A full description of the statistical parameters including central tendency (e.g. means) or other basic estimates (e.g. regression coefficient) AND variation (e.g. standard deviation) or associated estimates of uncertainty (e.g. confidence intervals)
☐	☒	For null hypothesis testing, the test statistic (e.g. F, t, r) with confidence intervals, effect sizes, degrees of freedom and P value noted<br>*Give P values as exact values whenever suitable.*
☒	☐	For Bayesian analysis, information on the choice of priors and Markov chain Monte Carlo settings
☒	☐	For hierarchical and complex designs, identification of the appropriate level for tests and full reporting of outcomes
☒	☐	Estimates of effect sizes (e.g. Cohen's d, Pearson's r), indicating how they were calculated

Our web collection on statistics for biologists contains articles on many of the points above.

Software and code

Policy information about availability of computer code

Data collection	Confocal imaging data: Leica TCS SP8 microscope using a 20X or 63X glycerin-immersion lens, with or without digital zoom.
Data analysis	Prism 6 from Graphpad was used for statistic analysis. Image (NIH image) was used for IHC and OHC counting. Canvas X GIS 2019 was used to composite images showing the whole cochlea.

For manuscripts utilizing custom algorithms or software that are central to the research but not yet described in published literature, software must be made available to editors and reviewers. We strongly encourage code deposition in a community repository (e.g. GitHub). See the Nature Portfolio guidelines for submitting code & software for further information.

Data

Policy information about availability of data

All manuscripts must include a data availability statement. This statement should provide the following information, where applicable:
- Accession codes, unique identifiers, or web links for publicly available datasets
- A description of any restrictions on data availability
- For clinical datasets or third party data, please ensure that the statement adheres to our policy

High-throughput sequencing data have been deposited in the NCBI Sequence Read Archive database under accession code SUB13659056. All other data are available from the corresponding authors on reasonable request.

Research involving human participants, their data, or biological material

Policy information about studies with human participants or human data. See also policy information about sex, gender (identity/presentation), and sexual orientation and race, ethnicity and racism.

Reporting on sex and gender	No human subjects were used.
Reporting on race, ethnicity, or other socially relevant groupings	NA
Population characteristics	NA
Recruitment	NA
Ethics oversight	NA

Note that full information on the approval of the study protocol must also be provided in the manuscript.

Field-specific reporting

Please select the one below that is the best fit for your research. If you are not sure, read the appropriate sections before making your selection.

☒ Life sciences ☐ Behavioural & social sciences ☐ Ecological, evolutionary & environmental sciences

For a reference copy of the document with all sections, see nature.com/documents/nr-reporting-summary-flat.pdf

Life sciences study design

All studies must disclose on these points even when the disclosure is negative.

Sample size	Same size calculation for hearing rescue were calculated based on power calculation or based on similar studies published by other groups. Sample size is given throughout the manuscript.
Data exclusions	For ABR measurements, a small fraction of frequencies for which thresholds were not apparent were excluded to ensure that only ABR thresholds scored accurately were used.
Replication	All experiments were successfully replicated, and represented results from at least three independent experiments on different days. For quantitative experiments a bigger sample size was selected where possible. We specify the number of biological replicates in the respective figure legends and in Statistics and reproducibility section
Randomization	Animals of the same genotypes (Atp2b2 Obi/+, C3H or Atp2b2 Obi/+: Tmcl Bth/+) were randomly randomly chosen be in the experimental groups in this study. Comparisons between the controls (untreated) and experimental (treated with lipo-RNP) was made based on genotype and whether animals were injected with RNP or not. Animals were raised under the same lighting, housing, and feeding conditions, and were on the same genetic background respectively . Both sexes of animals were used in each experiment and we did not observe any difference in the distribution of males/female in each genotype. Randomization was not a factor in the experiments, except for those involving animals in this study.
Blinding	All data from in vivo experiments were collected and analyzed by at least two researchers who were both blinded to experiment.

Reporting for specific materials, systems and methods

We require information from authors about some types of materials, experimental systems and methods used in many studies. Here, indicate whether each material, system or method listed is relevant to your study. If you are not sure if a list item applies to your research, read the appropriate section before selecting a response.

Materials & experimental systems

n/a	Involved in the study
☐	☒ Antibodies
☐	☒ Eukaryotic cell lines
☒	☐ Palaeontology and archaeology
☐	☒ Animals and other organisms
☒	☐ Clinical data
☒	☐ Dual use research of concern
☒	☐ Plants

Methods

n/a	Involved in the study
☒	☐ ChIP-seq
☒	☐ Flow cytometry
☒	☐ MRI-based neuroimaging

Antibodies

Antibodies used

Immunofluorescence: mouse anti-Parvalbumin (Sigma P3088), rabbit anti-pmca2 (PAl-915 ThermoFisher scientific), donkey anti rabbit Alex488 (A21206, ThermoFisher scientific), donkey anti- mouse Alex594 (A32744, ThermoFisher scientific). All of them have been used by others and by us in previously published studies on the adult mouse cochlea.

Validation

The anti-Parvalbumin (Sigma P3088), anti-pmca2 (PAl-915 ThermoFisher scientific), anti rabbit Alex488 (A21206, ThermoFisher scientific), anti- mouse Alex594 (A32744, ThermoFisher scientific), have been validated and is used routinely for staining hair cells. Hair cell morphology can be easily recognized in the confocal images

Eukaryotic cell lines

Policy information about cell lines and Sex and Gender in Research

Cell line source(s)

Atp2b2Obl/+ and Atp2b2Obl/Obl mouse primary fibroblasts were obtained from P4 pups. HEI-OC1 cells (RRID:CVCL_D899) were a gift from Dr. Albert Edge, Mass Eye & Ear/Harvard Medical School. Obl-OCl cells were created in the lab

Authentication

HEI-OC1 Cells were authenticated by the supplier, no further authentication was done for cell lines. Primary cells were generated in the lab and authentication was done by genotyping.

Mycoplasma contamination

All cell lines tested negative for mycoplasma contamination

Commonly misidentified lines
(See ICLAC register)

The study did not involve commonly misidentified lines.

Animals and other research organisms

Policy information about studies involving animals; ARRIVE guidelines recommended for reporting animal research, and Sex and Gender in Research

Laboratory animals

Mouse strains used in this study:
C3HeB/FeJ (C3H),
heterozygous Atp2b2 Obi/+ , in C3H background
homozygous Atp2b2 Obi/Obi, in C3H background
Pmca2Obl/+Tmc1Bth/+, in C3H background
Mice of either sex at PO-PS were used for culture and injection. Acoustic tests were performed 1-4 months after the injection.
The mice were housed at the animal facility of the Mass Eye and Ear Infirmary in a 12-hour-light/12-hour-dark cyclic environment.
The temperature ranged from 73°F to 76°F, and the humidity from 30% to 40%.

Wild animals

The study did not use wild animals

Reporting on sex

The study did not take into account the sex of the mice used.

Field-collected samples

The study did not involve samples collected in the field.

Ethics oversight

All in vivo experiments were carried out in accordance with NIH guidelines for the care and use of laboratory animals and were approved by the Massachusetts Eye & Ear Infirmary IACUC committee.

Note that full information on the approval of the study protocol must also be provided in the manuscript.
